# Calcitriol Modulates Both the Vitamin D Receptor and the Calcium-Sensing Receptor in Blood Mononuclear Cells in Elderly Female Patients with Hip Osteoporotic Fractures

**DOI:** 10.3390/biom16020266

**Published:** 2026-02-08

**Authors:** Javier Caballero-Villarraso, Ainoa Navarrete-Pérez, Antonio Camargo, Leo Valentín-Aragón, José Luis Gómez-Chaparro, José Manuel Quesada-Gómez, Antonio Casado-Díaz

**Affiliations:** 1 Maimónides Biomedical Research Institute of Córdoba (IMIBIC), Av. Menéndez Pidal, S/N, 14004 Córdoba, Spain; ep2napea@uco.es (A.N.-P.); antonio.camargo@imibic.org (A.C.); z22vaarl@uco.es (L.V.-A.); b22gomoj@uco.es (J.L.G.-C.); md1qugoj@uco.es (J.M.Q.-G.); 2 Clinical Analyses Service, Reina Sofía University Hospital, 14004 Córdoba, Spain; 3 Department of Biochemistry and Molecular Biology, University of Córdoba, 14071 Córdoba, Spain; 4 Lipids and Atherosclerosis Unit, Department of Internal Medicine, Reina Sofía University Hospital, 14004 Córdoba, Spain; 5 Biomedical Research Networking Centre (CIBER) Fisiopatología de la Obesidad y Nutrición (CIBEROBN), Instituto de Salud Carlos III, 28029 Madrid, Spain; 6 Carlos Castilla del Pino Health Centre, C/ Isla Lanzarote, S/N, 14011 Cordoba, Spain; 7 Endocrinology and Nutrition Department, Reina Sofía University Hospital, 14004 Córdoba, Spain; 8 Department of Nursing, Pharmacology and Physiotherapy, University of Córdoba, 14004 Córdoba, Spain; 9 Biomedical Research Networking Centre (CIBER) de Fragilidad y Envejecimiento Saludable (CIBERFES), Instituto de Salud Carlos III, 28029 Madrid, Spain

**Keywords:** vitamin D receptor, calcium-sensing receptor, calcidiol, 25(OH)D, calcitriol, l,25(OH)_2_D_3_, apoptosis, peripheral blood mononuclear cells

## Abstract

Introduction: Peripheral blood mononuclear cells (PBMCs) constitute a diverse population of cells involved in adaptive and innate immunity, playing an essential role in pathogen recognition, immune signaling, and immune response modulation. Vitamin D deficiency through the regulation of vitamin D receptor (*VDR*) and calcium-sensing receptor (*CaSR*) gene expression could influence the apoptotic functioning of PBMCs, which, despite its importance in the immune response, has not been sufficiently explored. Objectives: This research aimed to detect differences in the mRNA expression of *CaSR*, *VDR*, and apoptosis of PBMcs between elderly women with hip fractures and vitamin D deficiency and healthy young women, as well as in older women both at baseline and after administration of calcitriol. Methods: A case–control study involving 44 women (22 and 20, respectively) was conducted. The case group (hip fracture) was administered 2 µg/day of calcitriol for two weeks and a before-and-after comparison was made. The baseline gene expression of *VDR* and *CaSR* in PBMCs, as well as the effects of calcitriol on both the *VDR*/*CaSR* regulation and PBMC apoptosis, were studied in both groups. Serum bone biomarkers were also assessed. Results: No differences were observed in creatinine and calcium serum levels between the young and elderly osteoporotic women studied. Serum phosphorus and 25-hydroxyvitamin D (25(OH)D) were low in osteoporotic fractured women with vitamin D deficiency. In contrast, intact parathyroid hormone (PTH_1–84_) and alkaline phosphatase were high, while no significant difference in calcitriol [l,25(OH)_2_D_3_] serum levels was observed. In elderly women, serum calcium, phosphorus, alkaline phosphatase, 25(OH)D, and calcitriol remained unchanged after intravenous calcitriol therapy; however, PTH_1–84_ decreased after the treatment. In comparison to the young women, the elderly women showed decreased *VDR* and increased *CaSR* mRNA expression in PBMCs, as well as higher monocyte apoptosis. Conclusions: Calcitriol administration increased both *VDR* and *CaSR* mRNA expression in PBMCs and decreased PBMC apoptosis. Conclusions: The results obtained support the role of the vitamin D endocrine system as a regulator of the immune response and thus may contribute to explaining certain aspects of the immune dysfunction reported in individuals with vitamin D insufficiency.

## 1. Introduction

The vitamin D endocrine system (VDES), traditionally recognized for its role in calcium homeostasis and bone metabolism, has emerged as a key regulator of the immune system. Its immunomodulatory effects are mainly exerted through its active metabolite, 1,25-dihydroxyvitamin D (1,25(OH)_2_D or calcitriol), which acts on the vitamin D receptor (*VDR*), a member of the nuclear receptor superfamily which is widely expressed in immune cells such as monocytes, macrophages, dendritic cells, and activated T and B lymphocytes, where it exerts important immunomodulatory and antiproliferative properties [1,2,3,4,5,6,7].

Vitamin D_3_ is produced in the skin from 7-dehydrocholesterol by UV irradiation or is nutritionally provided through diet. The liver and other tissues metabolize vitamin D into 25-hydroxyvitamin D (25(OH)D), the main circulating form of vitamin D and the cornerstone of the VDES. 25-Hydroxyvitamin D is subsequently metabolized to 1,25(OH)_2_D by the CYP27B enzyme; this mainly occurs in the kidney, although other tissues, including the parathyroid gland, various epithelial cells, and cells of the immune system, also contain this enzyme [1]. The expression of the CYP27B1 enzyme in immune cells allows for the local production of calcitriol, which enables autocrine and paracrine regulation of the immune response [1,2].

Vitamin D (25(OH)D) deficiency is perhaps the most common nutritional deficiency in the world, which particularly affects many at-risk groups [8]. Organizations and scientific societies worldwide have published guidelines for recommended desirable 25(OH)D serum levels and vitamin D intake [9], but the minimal thresholds of 25(OH)D across age groups and populations are still debated [10]. Nevertheless, a consensus on the definition of sufficiency, insufficiency, or deficiency of vitamin D, as well as desirable serum 25(OH)D levels, has not yet been reached [11,12]. Severe deficiency is commonly defined as <10 ng/mL, deficiency as 10 to 20 ng/mL, and insufficiency is defined as 20–29.99 ng/mL, with levels between 30 and 50 ng/mL could be considered adequate and safe [13].

Elderly female patients who have suffered fractures and undergone surgery typically present severe vitamin D deficiency [14,15,16,17,18,19]. Aging causes a decrease in serum levels of 25-hydroxyvitamin D through various mechanisms (e.g., decreased vitamin D synthesis, decreased intestinal absorption) [20]. Immobility indoors and inflammation resulting from surgery, which worsens the inflammatory status of the elderly, are factors that contribute significantly to the decrease in 25-hydroxyvitamin D levels observed in older women undergoing surgical treatment for hip fractures, such as those who participated in this study [20,21]. As prolonged vitamin D deficiency is a determining factor in the development of osteoporosis, an adequate level of 25(OH)D must be maintained regardless of age, in order to prevent increased bone turnover and loss of bone quantity and quality [14,15,16,17,18,19].

The relative impact that vitamin D deficiency could have on both calcitriol autocrine and paracrine actions may be a factor that has not yet been sufficiently considered, which can mediate the alterations in immune response, cellular growth and differentiation, and various organ and tissue functions. Vitamin D deficiency associated with aging is one of the causes contributing to the negative regulation of *VDR*, leading to a less effective immune response [15,16,17,22]. The regulation of *VDR* expression is an important mechanism modulating the responsiveness of target tissues to calcitriol; in fact, the biological activity of calcitriol in cells is directly proportional to the tissue *VDR* concentration [23,24,25]. *VDR* expression is modulated by 1,25(OH)_2_D_3_ and several other hormones (including retinoic acid glucocorticoids and estrogen). The gene expression of *VDR* is also influenced by various physiological states such as age, pregnancy, lactation, and dietary calcium restriction. It has been shown that vitamin D deficiency decreased *VDR* mRNA levels in all the tissues studied [24,25,26]. Furthermore, the expression of *VDR* mRNA was decreased by vitamin D deficiency in PBMCs from elderly women but increased and returned to control values after intravenous calcitriol treatment [27,28]. Vitamin D signaling through the *VDR* involves genomic (modulation of immunoregulatory gene transcription) and non-genomic (rapid signaling through membrane and cytoplasmic *VDR*) mechanisms [5]. Interaction with the calcium-sensing receptor (*CaSR*) and regulation of mineral homeostasis influence the activation of intracellular signaling pathways, such as mTOR and autophagy, which are relevant to the function of monocytes and lymphocytes [6].

Peripheral blood mononuclear cells (PBMCs) are a diverse population of immune cells readily accessible, including both innate immune cells responsible for rapid pathogen responses and adaptive immune cells involved in specific immune responses. PBMCs play an essential role in pathogen recognition, immune signaling, and modulation of the immune response and express *VDR* and are thus targets for the immunomodulatory actions of calcitriol [29,30]. PBMCs have been shown to present *CaSR*, initially cloned from bovine parathyroid glands [31]. The main function of *CaSR* is to regulate bone and mineral metabolism by influencing parathyroid hormone secretion, urinary calcium excretion, and bone remodeling; however, there are expressed ubiquitously in the body, exerting pleiotropic actions in cells, including modification of proliferation, differentiation, and programmed cell death (apoptosis) [29,32,33]. *CaSR* may act as an immunomodulator in PBMCs depending on vitamin D, as well as in the ‘reversal’ phase of bone remodeling [31,33].

Vitamin D deficiency through the regulation of vitamin D receptor (*VDR*) and calcium-sensing receptor (*CaSR*) expression, in addition to influencing intestinal calcium absorption [15,34,35] and immune responses [25,36,37], could influence the apoptotic functioning of PBMCs, which, despite its importance in the immune response, has not been sufficiently studied [38,39].

This research aimed to detect differences in the mRNA expression of *CaSR*, *VDR*, and apoptosis of PBMCs between elderly women with hip fractures and a vitamin D-deficient status and healthy young women, as well as in elderly women both at baseline and after administration of calcitriol.

## 2. Materials and Methods

### 2.1. Subjects and Procedures

Blood samples were obtained at baseline from 20 young women as a control group (mean age: 33 years) and from 22 elderly women with a history of hip fractures (mean age: 75 years) in the previous three months (considered as a strict inclusion criterion), both before and after calcitriol treatment (intravenous administration of 2 µg calcitriol (Calcijex^®^, Roche, Basel, Switzerland) daily for two weeks). Exclusion criteria were that at the time of the study, the participants were not suffering from diabetes mellitus or intercurrent infections, malabsorption, nephrolithiasis, primary hyperparathyroidism, hyperthyroidism, hypercalcemia, creatinine clearance < 30 mL/min, neoplastic disease within the last 5 years and were not undergoing treatment with vitamin D metabolites or analogs or drugs that can modify vitamin D levels, calcium channel blockers, calmodulin antagonists, steroids, or immunosuppressive therapy.

Authorization for the study was obtained from the Biomedical Research Ethics Committee of the province of Córdoba. All participating women signed the informed consent form. The harmonized tripartite standards of the Declaration of Helsinki, the Organic Law on Biomedical Research of 15/2007 of 3 July, the Organic Law on Personal Data Protection (LOPD) of 13 December 2018, the code of ethics of the “Organización Médica Colegial” (OMC), the basic regulatory law 41/2002 on patient autonomy and rights and obligations regarding clinical information and documentation, of 14 November, and the standards of good clinical practice were respected.

### 2.2. Serum Parameters Analysis

Total calcium, serum phosphorus and alkaline phosphatase were measured using an autoanalyzer ADVIA Centaur XP (Siemens Healthineers, Erlangen, Germany). Serum PTH_1–84_ was determined via the allegro immunoradiometric assay from the Nichols Institute (San Juan Capistrano, CA, USA). Vitamin D status was assessed by measuring the level of [25(OH)D] with a chemiluminescence autoanalyzer Architect *c*-16000 (Abbott, Chicago, IL, USA), and 1,25(OH)_2_D_3_ (calcitriol) was determined via radioimmunoassay with an RIA kit (Immunodiagnostic systems Ltd., Boldon, UK). According to the serum levels, a 25(OH)D deficiency was defined as <20 ng/mL, insufficiency was defined as 20–29.99 ng/mL, and a normal level was defined as >30 ng/mL [22,23].

### 2.3. Apoptosis Measurement

PBMCs were separated by centrifugation on a Ficoll-Hypaque density gradient. The percentage of apoptotic cells was quantified as those positive for propidium iodide, as it can enter the nucleus and bind to the DNA of cells with a broken membrane. Apoptosis was assessed via flow cytometry with fluorescence-activated cell sorting (FACScan) (Beckton Dickinson, San Jose, CA, USA). Briefly, cells were washed and fixed in 70% ethanol for 3 h at 4 °C. Then, the cells were resuspended in 0.5 mL PBS to which 0.5 mL of RNase solution (l mg/mL) and propidium iodine (50 mg/mL) were added. Apoptotic cells were characterized by the ‘hypodiploid’ peak on flow cytometric analysis [40,41].

### 2.4. Gene Expression Quantification

Total RNA was isolated with Trizol Reagent from Invitrogen (Carlsbad, CA, USA). and the amounts of *VDR* and *CaSR* mRNA were measured via quantitative RT-PCR (qRT-PCR). The quantification of different amplicons was accomplished via laser-induced fluorescence with the ABI 373 A Stretch Sequencer from Applied Biosystems (Foster City, CA, USA) [42,43].

The primers for *VDR* were 5′ TGAAGGCTGCAAAGGCITCTTCAGGC 3′ (forward) and 5′ GGATGAACTCCITCATCATGCCGATG 3′ (reverse); those for *CaSR* were 5′ ATTGAGGGGGAGCCCACCTGCTGCT 3′ (forward) and 5′ AAAGAGGGTGAGTGCGATCCCAAAGG 3′ (reverse); and those for actin (housekeeping gene used for normalization) were 5′ CGTCACCAACTGGGACGACATGGAG 3′ (forward) and 5′ GGCGTACAGTAGCACAGCCTGGA 3′ (reverse).

### 2.5. Statistical Study

Data were analyzed using the Sigma statistical package for microcomputers from Horus (Madrid, Spain). The Shapiro–Wilk test was performed to check whether the values of each variable followed a normal distribution. As this was not the case, non-parametric tests were performed. To compare means in the case–control study, a Mann–Whitney U test was performed, and in the before-and-after study, a Wilcoxon signed rank test was performed (i.e., to compare *VDR* and *CaSR* gene expression values) within each group before and after calcitriol administration). All values are expressed as mean ± standard deviation. Statistical significance was accepted for *p* < 0.05.

## 3. Results

The 22 elderly women admitted for hip fractures did not differ from the young women in the control group in terms of ethnicity or anthropometric measurements (weight, abdominal circumference), etc. Before treatment with calcitriol, various parameters were compared between the group of young women and the group of older women with fractures.

No significant differences were observed in creatinine, calcium, and calcitriol (1,25(OH)_2_D) serum levels between the two groups. However, serum phosphorus (*p* < 0.05) and 25(OH)D (*p* < 0.001) were significantly decreased, whereas PTH_1–84_ (*p* < 0.0001) and alkaline phosphatase (*p* < 0.001) were significantly higher in older women (Table 1). As shown in the table below, none of the groups of women studied had sufficient levels of vitamin D (25(OH)D > 30 ng/mL). Thus, the mean serum 25(OH)D level in the group of elderly women with fractures was 8.0 ± 4.5 ng/mL, indicating a more severe vitamin D deficiency. In the group of young women, the mean value was between 20 and 30 ng/mL, which is considered insufficient.

In elderly osteoporotic women, total serum calcium (9.7 ± 0.4 mg/dL), phosphorus (2.5 ± 0.7 mg/dL), alkaline phosphatase (67 ± 18 UI/L), 25(OH)D_3_ (7 ± 4.8 ng/mL), and calcitriol (41 ± 12.0 pg/mL) remained stable after intravenous calcitriol therapy; in contrast, PTH_1–84_ decreased significantly after calcitriol treatment (46 ± 6 ng/mL; *p* < 0.001).

**Figure 1:**
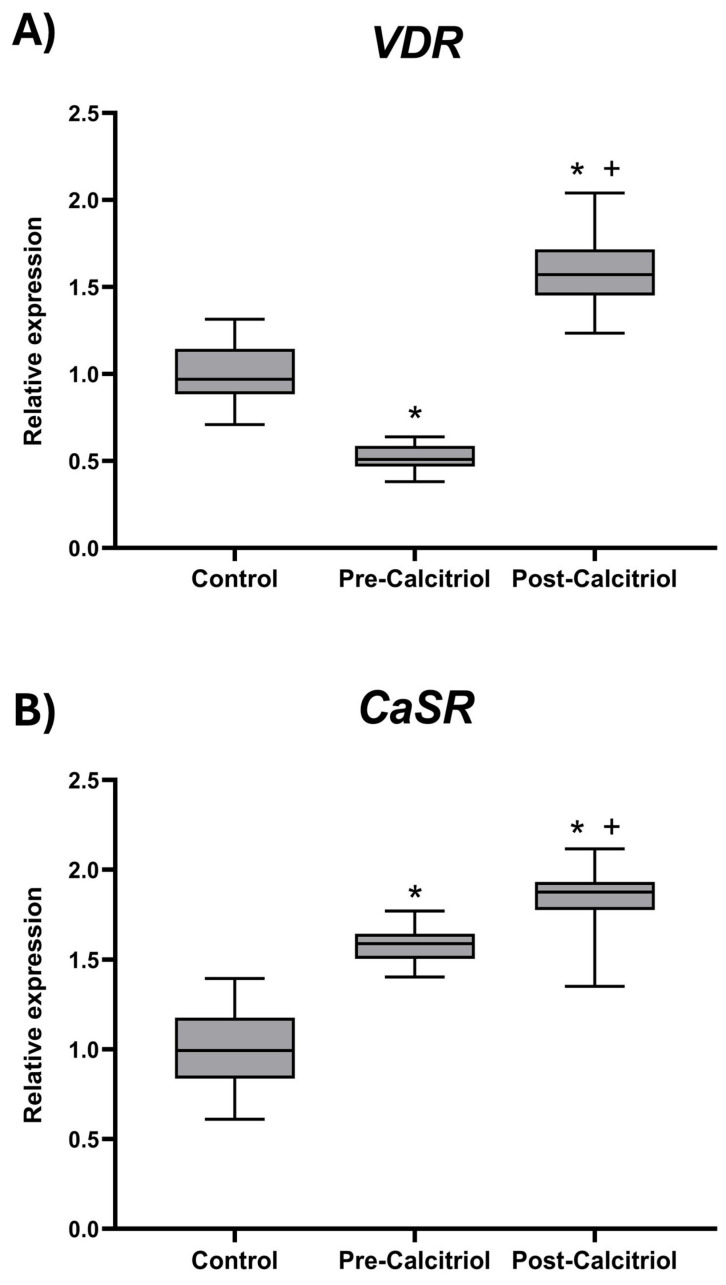
Expression levels of *VDR* (**A**) and *CaSR* (**B**) coding genes. Total RNA was isolated from PBMCs derived from the women included in the study. The mRNA levels of the *VDR* and *CaSR* genes were quantified in each of the RNA samples obtained from the control group (n = 20, young women) and the group of elderly women (n = 22) before (Pre-calcitriol) and after (Post-calcitriol) treatment with calcitriol. The graphs show the relative expression of each gene for each of the study groups. * *p* < 0.05 vs. Control; + *p* < 0.05 vs. Pre-calcitriol. The notches in each group indicate a 95% confidence interval (CI).

**Figure 2:**
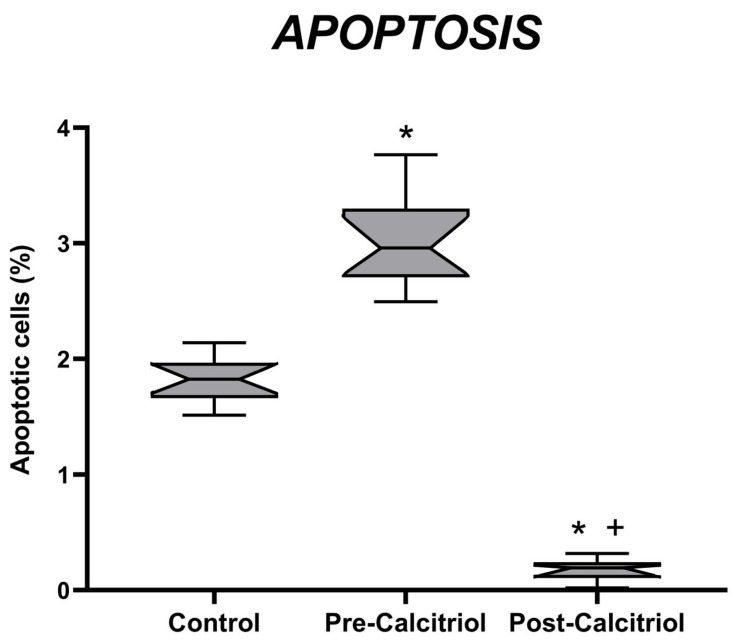
Quantification of monocyte apoptosis in PBMCs derived from young women (n = 20) and elderly women (n = 22), before (Pre-calcitriol) and after (Post-calcitriol) treatment with calcitriol. The percentage of apoptotic monocytes was determined in each sample. * *p* < 0.05 vs. Control; + *p* < 0.05 vs. Pre-calcitriol. The notches in each group indicate a 95% confidence interval (CI).

As shown in Figure 1, elderly osteoporotic women with vitamin D deficiency presented decreased *VDR* (*p* < 0.05) and increased *CaSR* (*p* < 0.05) mRNA expression levels in PBMCs, in comparison with the control group of women, as well as a higher PBMC apoptosis level (Figure 2). After treatment with calcitriol, in the group of elderly women, an increase in *VDR* (*p* < 0.05) and *CaSR* (*p* < 0.01) mRNA expression in PBMCs was observed, compared to pre-treatment values and compared to those of the control group (Figure 1).

The percentage of apoptotic cells was also studied in PBMCs. Before treatment with calcitriol, the group of elderly women had a higher percentage of apoptotic cells than the control group. However, after treatment with calcitriol, the percentage of apoptosis in elderly women decreased significantly. It was more than 14 times lower than the values obtained before treatment and more than 8 times lower than the values in the control group (Figure 2).

## 4. Discussion

The results of this study demonstrated that all elderly women with hip fractures who underwent surgery had serum 25(OH)D levels in the deficiency range, lower than those of young women in the control group, but higher levels of PTH1–84 in the secondary hyperparathyroidism range. In addition, they had lower expressions of *VDR* and *CaSR* and increased apoptosis in PBMCs. A short course of intravenous calcitriol treatment every 24 h in these patients induced an upregulation of *VDR* and *CaSR*, as well as a marked decrease in apoptosis in PBMCs.

The results of the present study also confirm the previously reported higher expression of *CaSR* mRNA in monocytes from osteoporotic women with vitamin D insufficiency, when compared to that in the controls [31,33]. These patients also showed an increase in monocyte *CaSR* mRNA expression after intravenous calcitriol treatment, which induced a significant decrease in serum PTH_1–84_ without an increase in serum calcium [44,45].

The current results also reveal higher expression of *CaSR* mRNA in PBMCs from osteoporotic elderly women with hip fractures and severe 25(OH)D deficiency, compared to those from young women. These patients also showed an increase in *CaSR* mRNA expression in monocytes after intravenous treatment with calcitriol, which induced a significant decrease in serum PTH_1–84_ without a concomitant increase in serum calcium [44,45].

Calcium is the main regulator of *CaSR*; therefore, extracellular calcium participates in the modulation of the immune response, possibly acting through transmembrane *CaSR* in mature monocytes/macrophages [46,47]. This upregulation of *CaSR* expression has previously been described in vitro in the parathyroid glands (PTG) and kidney [48]; as well as in HL-60 cells, which occurred during their differentiation into cells with a monocyte/macrophage phenotype in response to calcitriol treatment.

The increases in *VDR* and *CaSR* induced by calcitriol might contribute to the decrease in monocyte apoptosis either directly, through changes in cell monocyte cycle progression [30], or indirectly, through the inhibition of HLA-DR expression [49,50] or a change in the pattern of cytokines secreted by the immune system in response to calcitriol [51,52]. This occurs because calcitriol diminishes the expression of type II HLA antigens (DR, DP and DQ) on monocytes [53,54], as well as inhibiting and/or exerting modulating actions on cytokines [55,56].

Increased expression of *VDR* and *CaSR* interacting together may decrease PBMCs apoptosis, allowing them to fight pathogens for a longer period, while modifying the secretion of proinflammatory cytokines through the nuclear factor κB (NF-κB) signaling pathway to modify the inflammatory response [30,51,52]. These findings suggest that stimulation of *CaSR* via *VDR* could be exploited to regulate the immune system through monocyte and macrophage activities and reduce inflammatory damage; as such, it shows potential as a target for the prevention and treatment of inflammatory diseases [55,56].

Furthermore, the effects of *VDR* upregulation on the regulation of the innate and adaptive immune systems are extensive. Stimulation of *VDR* in monocytes (as well as macrophages, and neutrophils) induces the secretion of the antibacterial peptides cathelicidin and defensin, which play important roles in innate immune defenses due to their ability to lyse bacteria [1,57]. Serum levels of 25(OH)D are associated with the expression and functionality of Toll-like receptors (TLRs), especially those involved in viral responses [1]. The activation of innate immunity receptors, such as TLR2, increases the expression of *VDR*, 1α-hydroxylase, and cathelicidin, suggesting that the VDES plays a role in the innate immune response against bacterial pathogens [57]. *VDR* stimulation has been shown to inhibit T cell expansion and modulate cytokine expression with Th2 polarization [58], as well as to inhibit B lymphocyte differentiation and proliferation and immunoglobulin secretion. Other immunological effects attributed to *VDR* stimulation include dendritic cell maturation, decreased HLA class II expression, and enhanced antigen processing and presentation, which induce the production of more tolerogenic cytokines [57,59,60]. This evidence collectively supports the idea that vitamin D deficiency plays a role in immune system dysfunction [61,62,63,64].

The strength of this study lies in its simplicity, using an in vivo model of women with age-related osteoporosis and hip fracture using clinical blood samples. These women constitute a homogeneous cohort with vitamin D deficiency (measured as 25-hydroxyvitamin D). The administration of calcitriol, the hormone of the vitamin D endocrine system, in a short course, which does not produce an increase in blood calcium levels, allows us to clearly assess the biological response, showing that *VDR* stimulation, in addition to producing auto-upregulation, induces a clear increase in *CaSR* expression, producing a decrease in PBMCs apoptosis, which may contribute to improving the altered innate immune response and reducing the high rate of infections as a complication of the natural history observed in women with hip fractures and vitamin D deficiency [65].

The main weakness of this study lies in the absence of additional molecular and mechanical analyses to further clarify the relationship between clear *VDR*/*CaSR* signaling and PBMC apoptosis. Another weakness we could consider is that, as this is a single-center study, we have less external validity. If we had a larger sample size and greater ethnic diversity, the patients could have been stratified by race and then compared. Another aspect that should be considered in future studies is to carry out longer follow-up, in order to find out whether the changes observed are maintained over time.

## 5. Conclusions

Our present results support the increasing evidence that the vitamin D endocrine system plays a role not only as a regulator of calcium homeostasis and bone health, but also as a regulator of the immune response. Therefore, this study may contribute to explaining the immune dysfunction associated with vitamin D deficiency.

Furthermore, stimulation of the *VDR* by vitamin D (or its structural analogs) can be considered as a therapeutic target for the prevention and treatment of infectious, inflammatory, or autoimmune diseases.

## Figures and Tables

**Table 1:** Serum biochemical parameters in young women (as controls) vs. those in elderly women with osteoporosis and hip fractures who had severe 25(OH)D deficiency and mild secondary hyperparathyroidism.

Parameter	Young Women (Control)	Elderly Women (Hip Fractured)	*p* Value
Age (years)	32.6 ± 7.2	75.4 ± 8.0	*p* < 0.0001 *
Creatinine (mg/dL)	0.9 ± 0.1	1.0 ± 0.08	*p* < 0.4600
Calcium (mg/dL)	9.7 ± 0.4	9.6 ± 0.3	*p* < 0.4800
Phosphorus (mg/dL)	3.7 ± 0.3	2.4 ± 0.6	*p* < 0.0500 *
Alkaline phosphatase (UI/L)	52.5 ± 10.0	71.2 ± 14.0	*p* < 0.0001 *
PTH_1–84_ (ng/mL)	36.0 ± 8.0	64.0 ± 12.0	*p* < 0.0001 *
1,25(OH)2D (pg/mL)	45.0 ± 5.0	41.0 ± 12.0	*p* < 0.4100
25(OH)2D (ng/mL)	22.4 ± 5.3	8.0 ± 4.5	*p* < 0.0001 *

* Statistically significant difference.

## Data Availability

The original contributions presented in this study are included in the article. Further inquiries can be directed to the corresponding authors.

## Abbreviations

The following abbreviations are used in this manuscript:

VDES	Vitamin D endocrine system
*VDR*	Vitamin D receptor
*CaSR*	Calcium-sensing receptor
PBMCs	Peripheral blood mononuclear cells
